# Reducing disparities in kidney transplantation for Spanish-speaking patients through creation of a dedicated center

**DOI:** 10.1186/s12882-022-02879-4

**Published:** 2022-07-15

**Authors:** Madhura Pande, Monica Grafals, Katherine Rizzolo, Elizabeth Pomfret, Jessica Kendrick

**Affiliations:** 1 Division of Renal Diseases and Hypertension, University of Colorado Anschutz Medical Campus, Aurora, CO USA; 2 Colorado Center for Transplant Care, Research and Education (CCTCARE), Aurora, CO USA; 3 Division of Transplant Surgery, University of Colorado Anschutz Medical Campus, Aurora, CO USA

**Keywords:** Transplantation, Disparities, Hispanic Americans

## Abstract

**Introduction:**

Hispanic Americans receive disproportionately fewer organ transplants than non-Hispanic whites. In 2018, the Hispanic Kidney Transplant Program (HKTP) was established as at the University of Colorado Hospital (UCH). The purpose of this quality improvement study was to examine the effect of this culturally sensitive program in reducing disparities in kidney transplantation.

**Methods:**

We performed a mixed-methods analysis of data from 436 Spanish-speaking patients referred for transplant to UCH between 2015 and 2020. We compared outcomes for patients referred between 2015–2017 (*n* = 156) to those referred between 2018–2020 (*n* = 280). Semi-structured phone interviews were conducted with 6 patients per time period and with 6 nephrology providers in the Denver Metro Area. Patients and providers were asked to evaluate communication, transplant education, and overall experience.

**Results:**

When comparing the two time periods, there was a significant increase in the percentage of patients being referred (79.5% increase, p-0.008) and evaluated for transplant (82.4% increase, *p* = 0.02) during 2018–2020. While the number of committee reviews and number waitlisted increased during 2018–2020, it did not reach statistical significance (82.9% increase, *p* = 0.37 and 79.5% increase, *p* = 0.75, respectively. During patient and provider interviews, we identified 4 themes reflecting participation in the HKTP: improved communication, enhanced patient education, improved experience and areas for advancement. Overall, patients and providers reported a positive experience with the HKTP and noted improved patient understanding of the transplantation process.

**Conclusions:**

The establishment of the HKTP is associated with a significant increase in Spanish-speaking Hispanic patients being referred and evaluated for kidney transplantation.

## Introduction

Racial and ethnic disparities in kidney healthcare persist at every step leading to kidney transplantation in the United States. Specifically, Hispanic patients have less access to transplantation than non-Hispanic white patients. The disparity begins in primary care, as Hispanic patients are twice as likely as non-Hispanic white patients to lack a consistent healthcare provider that could refer them to nephrology [1]. These patients experience delays in receiving specialized kidney care and are 39% less likely than non-Hispanic whites to receive at least 12 months of pre-dialysis nephrology care [2]. Despite established kidney care, Hispanic patients are still less likely to be referred for transplantation compared to non-Hispanic white and Black patients [3]. Once initiated on dialysis treatment, Hispanic patients with kidney failure also experience a longer delay in becoming active on the waitlist [4]. Finally once listed, Hispanic patients experience longer waiting times for kidney transplantation than non-Hispanic whites [5]. The time from being diagnosed with end-stage kidney disease (ESKD) to deceased donor kidney transplantation (DDKT) is shorter for non-Hispanic whites, and this disparity increases when considering that Hispanic patients are younger and healthier when diagnosed with ESKD [6]. Furthermore, Hispanic patients undergo less live donor kidney transplantation (LDKT) than non-Hispanic whites, which has better outcomes than long term dialysis or DDKT [2].

A key strategy for reducing disparities in transplantation is the development of culturally competent programs. In 2018, the University of Colorado established the Hispanic Kidney Transplant Program (HKTP) as part of the Colorado Center for Transplant Care, Research and Education (CCTCARE) to reduce racial/ethnic disparities in kidney transplantation. In the HKTP, the providers, nursing staff, financial coordinator, social worker and support staff are all Spanish-speaking individuals well versed in the cultural differences affecting this population of patients. The HKTP performs pre-transplant evaluation clinics twice a week, waitlisting clinics once a week and post-transplant clinics at least twice a week. The purpose of this study was to assess the effect of this program in reducing barriers to kidney transplantation in Spanish-speaking only patients. Spanish-speaking only patients may be less acculturated than primarily English-speaking Hispanics and may have more barriers to kidney transplantation. We hypothesized that the HKTP would result in more Spanish-speaking only Hispanic patients undergoing evaluation, being placed on the waitlisting and ultimately receiving a kidney transplant.

## Methods

Utilizing data from University of Colorado Hospital, we identified all adults (aged 18 or older) that identified as Spanish-speaking who were referred for kidney transplantation or kidney-pancreas transplantation between January 1, 2015 and December 31, 2020. We examined the number of transplant evaluations, committee reviews, number of patients placed on the waiting list and the number receiving a kidney transplant. We compared these outcomes for patients referred between 2015–2017 to those referred between 2018–2020 after the establishment of the Hispanic Kidney Transplant Program.

We conducted semi-structured interviews with patients and providers to gain additional insight regarding kidney transplantation in the Spanish-speaking population. Interviews were conducted via phone by a trained interviewer (MP performed the Spanish-speaking patient interviews and JK performed the provider interviews). Interviews were conducted until thematic saturation was reached (i.e. no new information was heard) and were 20–30 min in duration. From previous experience, we expected to need to interview 5 to 6 patients and providers in order to reach saturation. We randomly selected 6 patients from each time period and 6 providers to interview and reached thematic saturation. Spanish-speaking patients from each time period (2015–2017 and 2018–2020) were included and were randomly selected (*n* = 12 total). Providers from the Denver Metro Area that refer patients to the University of Colorado for kidney transplantation were invited randomly by email. We included both nephrologists and advanced practice providers (APPs). The interview guide contained open-ended questions assessing communication, kidney transplant education and overall experience with the transplant center at the University of Colorado. Patients and providers completed a brief demographic questionnaire at the end of the interview. This study was performed in accordance with the Declaration of Helsinki and was approved and certified as exempt by the Colorado Institutional Review Board (COMIRB 20–2346). All participants gave consent by completing the interviews. The Consolidated Criteria For Reporting Qualitative Health Research (COREQ) was followed [7].

### Analysis

Standard descriptive statistics were used to examine the number of kidney transplant referrals, evaluations, committee reviews, number waitlisted and number receiving transplants. Pooled t-tests were used to examine differences in referrals, evaluations, committee reviews, number waitlisted and number receiving transplants between the two time periods. All analyses were done using SAS statistical software, version 9.4 (SAS Institute Inc., Cary, NC).

All interview data was analyzed using Thematic Analysis to elicit common themes. Two separate coders reviewed the data in parallel and identified key concepts, themes and ideas. The investigators then reviewed and discussed the main themes to reduce overlap and redundancy among the categories.

## Results

**Fig. 1:**
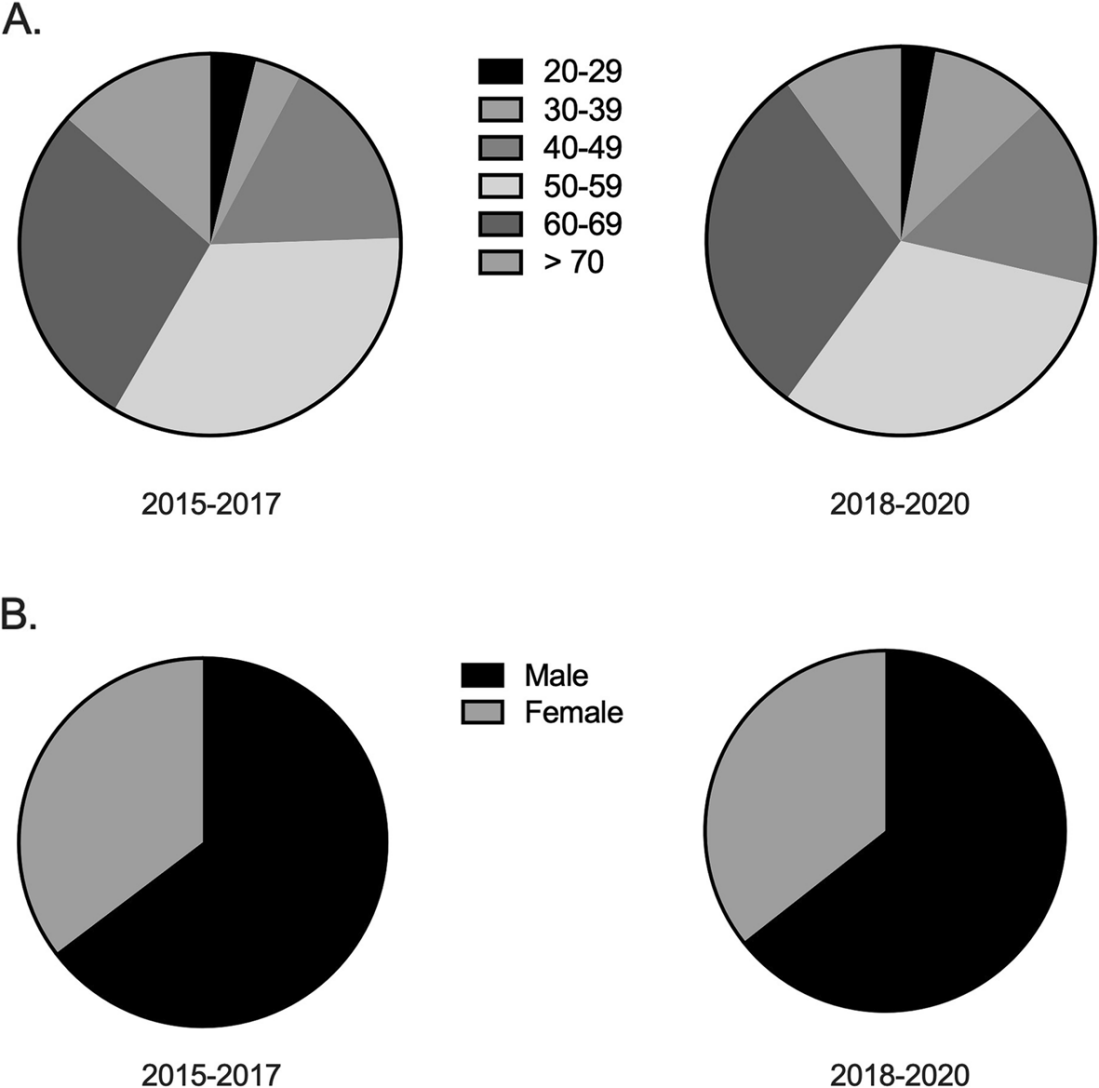
Demographic Characteristics of Patients at Transplant Referral. **A** Patient Age (Years) at Transplant Referral. **B** Patient Sex at Transplant Referral

**Fig. 2:**
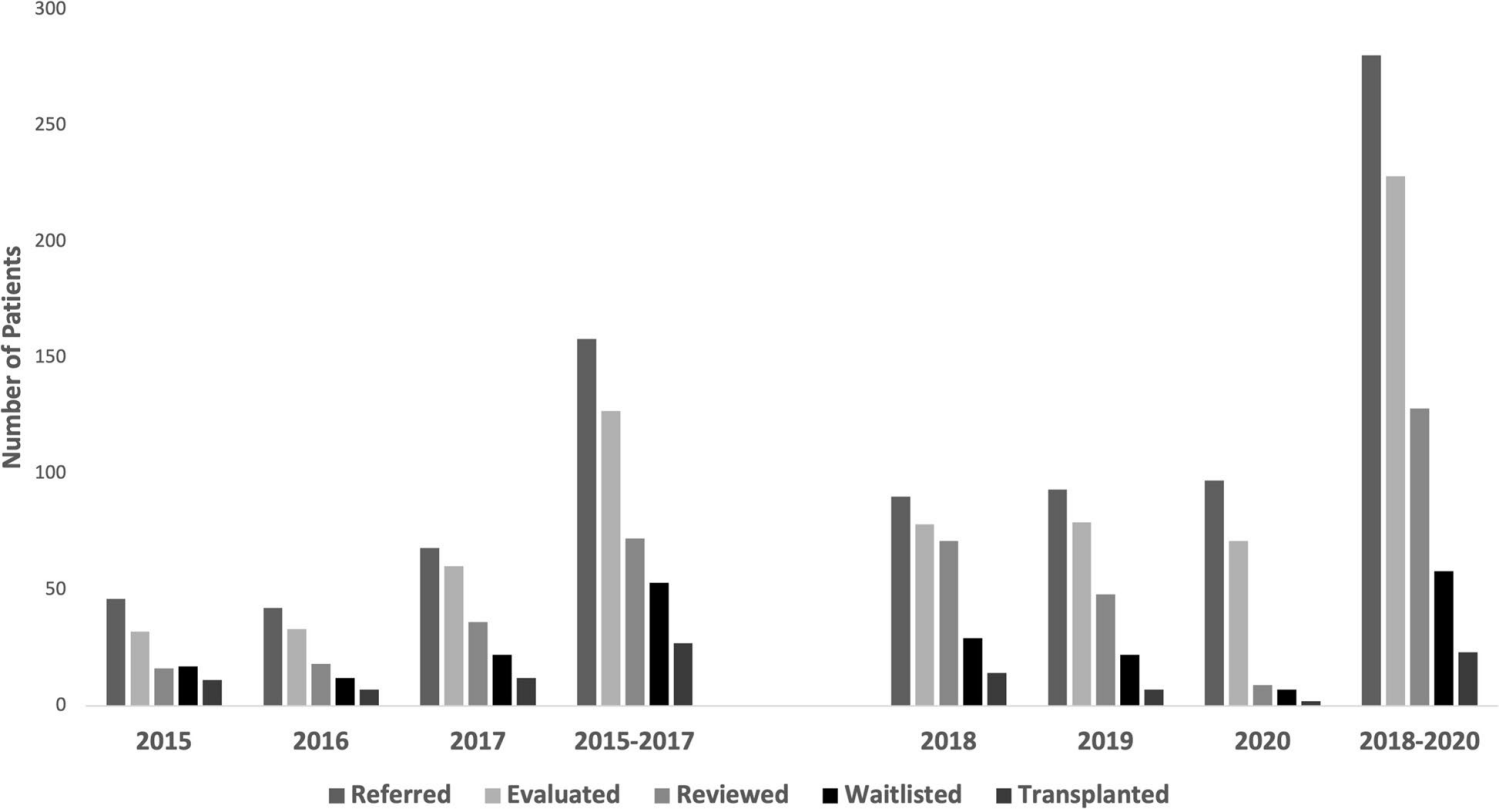
Transplant Outcomes of Spanish-Speaking Patients at the University of Colorado Hospital between 2015–2017 and 2018–2020

Four hundred thirty-six Spanish-speaking patients were referred for kidney transplantation at the University of Colorado between 2015 and 2020. One hundred fifty-six patients were referred between 2015–2017 compared to 280 between 2018–2020. Figure 1 shows baseline characteristics of the participants. The majority of patients were between the ages of 50–69 years and 64.4% were male. There were no significant differences in characteristics between the two time periods. Figure 2 shows transplant outcomes across each year and across pooled years from 2015–2017 and 2018–2020. Figure 2 shows the number of patients completing each step of the kidney transplantation process in each of the time periods. The number of patients referred, evaluated, reviewed and waitlisted increased in 2018–2020 compared to 2015–2017 (Table 1). Compared to 2015–2017, in 2018–2020 there was a statistically significant increase in the number of patients referred for transplant (79.4% increase, *p* = 0.008), Table 1. There was also a significant increase in the number of patients evaluated in 2018–2020 compared to 2015–2017 (82.4% increase, *p* = 0.02). While there was an increase in the number of patients undergoing a committee review (82.9% increase), and an increase in the number waitlisted (13.5% increase), these did not reach statistical significance. While it appears there was a decrease in the number of patients receiving a kidney transplant, the number of kidney transplants performed at UCH was significantly impacted in 2020 by the COVID-19 pandemic and there was no statistical difference between 2015–2017. When data from 2020 was excluded, there was a 5% increase in the number of Spanish-speaking patients transplanted and a 50% increase in the number of patients wait-listed, although these did not reach statistical significance (Table 2).

**Table 1:** Differences in transplant outcomes between 2015–2017 and 2018–2020

Outcome	Mean Annual Average (Number) 2015–2017	Mean Annual Average (Number) 2018–2020	Mean Difference in Annual Average (95% CI)	Percentage Increase from 2015–2017	*P*-value
Referred	52.0	93.3	-41.3 (-64.5 to -18.2)	79.4	0.008
Evaluated	41.7	76.0	-34.3 (-60.7 to -7.9)	82.4	0.02
Reviewed	23.3	42.7	-19.3 (-72.6 to 33.9)	82.9	0.37
Waitlisted	17.0	19.3	-2.3 (-22.0 to 17.4)	13.5	0.75
Transplanted	10.0	7.7	2.3 (-8.2 to 12.9)	-23.3	0.57

**Table 2:** Differences in transplant outcomes between 2015–2017 and 2018–2019 (excluding 2020)

Outcome	Mean Annual Average (Number) 2015–2017	Mean Annual Average (Number) 2018–2019	Mean Difference in Annual Average (95% CI)	Percentage Increase from 2015–2017	*P*-value
Referred	52.0	91.5	-39.5 (-72.9 to -6.1)	76.0	0.03
Evaluated	41.7	78.5	-36.8 (-74.5 to 0.8)	88.3	0.05
Reviewed	23.3	59.5	-36.2 (-73.9 to 1.6)	155.4	0.05
Waitlisted	17.0	25.0	-8.5 (-22.9 to 6.0)	50.0	0.16
Transplanted	10.0	10.5	-0.5 (-10.9 to 9.9)	5.0	0.89

Compared to the Organ Procurement and Transplantation (OPTN) Region and the rest of the United States, the University of Colorado has a higher percentage of Hispanic patients on the waiting list (Table 3). This includes new patients added in the year 2020 as well as all patients currently on the waiting list. Additionally, the number of Hispanic patients receiving a deceased donor kidney transplant in 2020 was higher than the OPTN region and the rest of the United States (30.4 vs. 11.0% for OPTN Region and 19.1% for the U.S.), Table 3.

**Table 3:** Number of Hispanics on Waiting List and Transplanted at UCH Compared to OPTN Region and U.S

	New waiting list registrations 1/1/2020 to 12/31/2020 (%)			All Waiting List Registrations on 12/31/2020 (%)			Deceased donor transplant recipient demographics for patients transplanted between 1/1/2020 and 12/31/2020 (%)			Living donor transplant recipient demographics for patients transplanted between 1/1/2020 and 12/31/2020 (%)		
	UCH *N* = 312	OPTN Region *N* = 1,937	U.S. (*N* = 37,653)	UCH *N* = 841	OPTN Region *N* = 3695	U.S. *N* = 97,493	UCH *N* = 184	OPTN Region *N* = 1254	U.S. *N* = 17,581	UCH *N* = 71	OPTN Region *N* = 276	U.S. *N* = 5,234
Hispanic	21.8	11.4	19.7	25.8	12.3	21.1	30.4	11.0	19.1	15.5	9.4	16.2

We conducted twelve patient interviews (six from each time period) from October 2020 through December 2020. Six men and six women were interviewed. Patient demographics are shown in Table 4. Six healthcare providers from the Denver Metro Area were interviewed, three MDs and 3 APPs. All of the providers had been in practice for over 4 years in the Denver Metro Area. All of the providers had experience referring patients to the University of Colorado Transplant Center before and after the development of the HKTP. Three were in private practice and three in academic practice. Four overall themes were identified: improved communication, enhanced patient education, improved patient experience and areas for advancement. Selected quotations from each theme are provided in Table 5.

**Table 4:** Demographic characteristics of the interviewed patients

Characteristic	2015–2017 (*n* = 6)	2018–2020 (*n* = 6)
Age (years)		
30–39	0 (0)	1 (16.7)
40–49	2 (33.3)	1 (16.7)
50–59	2 (33.3)	3 (50.0)
60–69	1 (16.7)	1 (16.7)
70 and older	1 (16.7)	0 (0)
Gender		
Female	3 (50.0)	3 (50.0)
Dialysis Vintage		
Not on dialysis	0 (0.0)	1 (16.7)
Less than 1 year	0 (0.0)	2 (33.3)
1–2 years	1 (16.7)	1 (16.7)
3–5 years	4 (66.6)	2 (33.3)
> 6 years	1 (16.7)	0 (0.0)

**Table 5:** Selected participant quotations for each theme identified

Theme	Quotations
Improved Communication	**2015–2017** “It was not good. The people did not speak Spanish.” (Man) “It was a bit difficult. I brought my grandson along to help me understand. I would not have been able to understand without him translating” (Woman)
	**2018–2020** “They took care of everything; even to this day they answer my calls” (Woman, HKTC patient) “They seem like a unified team, complete with social workers and all.” (Man, HKTC patient) “I had no issues. The doctor spoke Spanish” (Man, HKTC patient)
	**Providers** “Communication has been significantly improved with the Hispanic clinic compared to before” (MD) “I have a direct line of communication with the Medical Director of the clinic which is extremely helpful” (MD) “Still overwhelming for patients to schedule testing as they have to go through English speaking individuals” (APP)
Improved Patient Education	**2015–2017** “Well, I think I understand some of it because my grandson told me” (Woman) “Like I said, I did not understand what was the problem. I still do not know what is happening with the kidney, if I can still get one or not” (Man)
	**2018–2020** “I feel good. I know what medications to take and how to take them. And not to miss an appointment” (Woman) “I feel grateful. I’m definitely doing my part, also working on my health…they [clinic team] are orienting me and they gave me a book with all the instructions” (Man)
	**Providers** “Patients have a better education and are able to demonstrate understanding of the risks and benefits post-visit” (MD) “Patients do not know who to get to order tests [necessary for listing] or how to get them done” (APP)
Improved Patient Experience	**2015–2017** “It was not good..it was not good. I have been waiting for a long time on the list..still I don’t know. I have not heard from anyone in a long time” (Man) “I was a little afraid, in the whole process. I know I need a new kidney, but with the tests and appointments, it was too much. I didn’t feel comfortable” (Man)
	**2018–2020** “It was very good, of the best actually. We received excellent service there, better than I would have imagined. It seemed like my care was very important to them. Almost everyone spoke Spanish” (Woman) “It was very good, specifically the quality of the doctors. She [the doctor] was very capable, she explained everything to us step by step. The doctor that did my transplant was also very thorough and very kind” (Woman)
	**Providers** “The patient’s experience is so much better as they are seeing doctors who speak their language” (APP) “ All of my patients have had a very positive experience” (MD) “Much easier to refer Spanish speaking patients now and we have gotten a lot more patients evaluated in the last two years” (MD)
Areas for Improvement	**2015–2017** “They should give more information before the transplant…more information and opportunities on what to do before transplant” (Woman)
	**2018–2020** “The only barrier is that we don’t communicate with each other. The ones that can donate, our people don’t know to donate organs if we can. This is the problem: we need to educate our people and our families so we can complete the process…how can we educate others?”(Man)
	**Providers** “Having a direct option of choosing the Hispanic clinic in Epic would be very helpful” (MD) “A written guide in Spanish in 2^nd^ to 3^rd^ grade language explaining what tests are needed to be done and why. Not just a letter of these are the tests that need to be completed” (APP) “If the testing could be completed in 1–2 days this would help more patients complete the listing process” (MD)

### Improved communication

All interviewed patients seen after 2018 described the HKTP team as thorough and did not identify any issues with communication between themselves and the HKTP team. Several patients described that they felt important to the providers. “I didn’t have any issues. If there were ever problems or they have to reschedule, they call me and let me know and we move step by step.” In comparison, patients seen in 2015–2017 all discussed issues with suboptimal communication. All of these patients reported difficulty understanding their status in the transplant process. Additionally, 4 out of the 6 reported significant issues with language in the clinic. “It was a bit difficult. I brought my grandson along to help me understand. I would not have been able to understand without him translating.” “My daughter was there to translate for me…my daughter told me what they said. I feel for people who don’t have children that can come with them.” Several patients discussed issues with written communication being sent in English and difficulty making appointments.

All of the providers discussed that it was easier to refer their Spanish-speaking patients to the HKTP and that more patients were undergoing kidney transplant evaluations. Several providers also discussed that communication between the transplant center and the patients was significantly improved following the development of the HKTP. “The patients are better able to communicate with the schedulers” (MD). Two providers did discuss that while everyone in the HKTP speaks Spanish, this is not the case when patients are scheduling and seeing other providers at UCH to get the necessary tests in order to be listed. “A stress test is ordered and they call to schedule it with the patient. But these calls are usually in English and the patient does not call back to schedule because they are waiting for their daughter/son/grandchild to translate the message and then help them call back” (APP). Many of the providers felt that communication between the transplant center and the providers was overall improved with the HKTP. The providers all discussed they have a direct line of communication with the Medical Director of the HKTP which was extremely helpful.

### Enhanced patient education

All of the patients seen in the HKTP expressed understanding of the kidney transplantation process and gratitude for the pamphlets provided in Spanish. “I feel good because I know what medications to take and how to take them. And to not miss an appointment.” Patients seen at the transplant center during 2015–2017 did not express a good understanding of the transplant process. “They should give more information on what to do before transplant.” Several patients discussed how they had to rely on their child/grandchild for understanding the process. “Well, I think I understand some it because my grandson told me.” Many patients did not understand their transplant status and mistakenly had thought they were listed for transplant when they had not completed all of the testing. “Last time they told me I needed to do some tests or something, but I did not know what they needed me to do. So I did not go. They also did not tell me how much it costs.”

Providers all discussed that the patients had a better understanding of the risks/benefits of kidney transplant after being seen in the HKTP. “Patients have a better education and are able to demonstrate understanding of the risks and benefits post-visit” (MD). While all the providers noted the patient’s knowledge of transplant was improved with the creation of HKTP, they did discuss that patients still do not have a great understanding of the next steps for transplant. Providers discussed that health literacy is often low in their patients and “they need more education and more prompting to get the testing done” (APP).

### Improved patient experience

All of the patients reported a very positive experience at the HKTP. Patients discussed they felt important and connected to the providers. “We received excellent service there, better than I would have imagined. It seemed like my care was very important to them.” In contrast, patients seen in 2015–2017 did not have as positive of an experience, “It was not good..it was not good. I have been waiting for a long time on the list..still I don’t know. I have not heard from anyone in a long time.” Others felt like Spanish-speaking patients did not have priority and the transplant team focused more on money. “I feel like we Hispanics don’t have priority for transplants.” Patients discussed more frustration with the overall process of scheduling, being seen and then following-up with the transplant center in 2015–2017. “I was a little afraid, in the whole process. I know I need a new kidney, but with the tests and appointments, it was too much. I didn’t feel comfortable.”

All of the providers discussed that the patients report a positive experience at the HKTP, “experience is so much better as they are seeing doctors who speak their language.” The providers also discussed an overall positive experience with the HKTP, discussing that having a direct line of communication with the Medical Director was very beneficial and that the HKTP was helping to prioritize transplantation in their Hispanic patients.

### Areas for advancement

While the overall experience of the HKTP was very positive, the providers did note several areas that could still be improved. While communication was felt to be significantly improved with the HKTP, the providers felt quarterly meetings between the HKTP and the providers would be very helpful to further improve communication. Providers outside the University hospital system, also discussed how streamlining the necessary testing for transplantation into 1–2 days would help more patients complete the listing process. Several providers noted that this would help all their patients, not just the Spanish-speaking ones as significant delays occur when trying to get the necessary testing done at an outside facility. Providers also discussed that further education and communication with the patients regarding the tests needed for transplant is essential. One provider discussed using “a written guide in Spanish in 2^nd^ to 3^rd^ grade language explaining what tests are needed to be done and why. Not just a letter of these are the tests that need to be completed” (APP). Other providers discussed the importance of a patient advocate to help with scheduling, follow-up, etc. Finally, the providers discussed that having a direct option of choosing the HKTP in the electronic medical record would be helpful. Providers described that currently they can only place a referral to the UCH Transplant Center in general and have to write in the comments that the patient is Spanish-speaking or write in a request for the HKTP.

When the patients were asked if they could think of any way the HKTP could be improved all of the patients discussed that it was great. One patient discussed education sessions with friends, family and the Hispanic community to help increase living kidney donation. “The only barrier is that we don’t communicate with each other. The ones that can donate, our people don’t know to donate organs if we can. This is the problem: we need to educate our people and our families so we can complete the process…how can we educate others?”.

## Discussion

We found the creation of the HKTP resulted in an increased number of Spanish-speaking patients per year being referred and evaluated for transplant. We found that patient and provider overall experience with the HKTP was very positive. Communication and patient education were also improved with the creation of the HKTP. Our results suggest that clinics tailored to Spanish-speaking patients may be a means of reducing transplant disparities for Hispanic Americans.

Our results are similar to findings from previous studies. At Northwestern Medicine in Illinois, the creation of a HKTP in 2006 resulted in a significant increase in living donor kidney transplants in Hispanic patients (74% increase) [8]. Additionally, the number of Hispanics added to the waiting list grew by 91%. North Carolina created a specialized Hispanic/Latino clinic in 2019 and found that it significantly increased the number of patients being referred, evaluated, waitlisted and transplanted [9]. However, unlike our study, this study did not obtain qualitative data to gain insight into the patients and providers perceptions of the program. Additionally, we show that the number of Hispanic patients being placed on the waiting list and receiving a deceased donor kidney transplant is higher at our center than our OPTN region and the U.S. Furthermore, to our knowledge, our study is the first to only report data on Spanish-speaking only patients. Previous studies have focused on all Hispanics referred to a program. We focused on Spanish-speaking patients as this group may have more barriers to transplantation than English-speaking patients. Collectively these data suggest that culturally competent transplant programs can reduce disparities in kidney transplant.

Reducing racial/ethnic disparities in kidney transplantation is critical as racial/ethnic minorities comprise > 60% of all kidney transplant candidates [10]. Hispanics are the largest and fastest growing ethnic minority group in the United States [10]. Numerous studies have identified disparities and barriers to kidney transplantation [11]. Unfortunately, disparities exist in all components of the transplantation process. Patient-, physician/provider- and system-related barriers have all been identified [11]. Studies have found that medical eligibility (such as age and comorbidities) does not explain ethnic disparities in transplantation. In fact, Hispanics had a lower prevalence of medical barriers to transplantation than non-Hispanic whites at the time of dialysis initiation [12]. Thus, other barriers may be playing a significant role in transplantation disparities.

Lack of patient knowledge regarding kidney transplantation is a barrier to transplantation. A recent study from our institution found that Hispanics reported less understanding of the benefits and process of transplant compared to non-Hispanic whites [13]. Interventions for living donation educations- including mass media, [14] culturally competent websites [15], live education sessions [16], community health/language concordant advisors [17], and navigators [18]- have all shown to be beneficial in transplant education for patients and their potential donors. In our study, both patients and their providers felt the patient’s knowledge of transplant was significantly improved with evaluations occurring in the HKTP. Participants felt more knowledgeable about the transplant process and the risks/benefits associated with transplant. Despite this, providers discussed that many patients do not understand the necessary testing and next steps in the transplant process. Low health literacy is a problem for many patients eligible for kidney transplantation and thus tailored education sessions and tools written in the appropriate language and grade level are necessary to improve disparities in transplantation. In the HKTP, transplant education sessions are conducted in Spanish by a transplant physician and patients are given written materials in Spanish. Having a transplant physician lead the education sessions is critical as many Hispanics feel more comfortable with trusting a physician rather than another healthcare provider [19].

Improving interactions between patients and providers is essential to reduce disparities. In our center, the creation of a culturally competent, Spanish-speaking clinic improved communication between patients and providers. All of the patients interviewed discussed how they felt important to the providers and trusted them. This increased interaction may be why patients felt more knowledgeable regarding transplantation and why more patients completed the transplant listing process. In the US, greater than 30 million people are unable to speak the same language as their healthcare provider [19]. In the HKTP, the transplant coordinator, social worker and transplant physician all speak Spanish. Hispanics with bilingual physicians are less likely to miss follow-up appointments or have nonadherence to medications [20].

It should be noted that lack of insurance or underinsurance remains a significant barrier to transplantation. Patients with Medicaid and Medicare as their primary insurance are less likely to receive a kidney transplant compared to those with commercial insurance [21] and racial/ethnic minorities are more likely to have lower levels of health insurance coverage than whites [22]. National policies, such as improved insurance coverage, are necessary to address this.

While the HKTP was successful, providers still identified areas that could be improved. Having a direct option to choose the HKTP in the EMR when placing a transplant consult is a simple solution that may help patients get through the referral process quicker. We are currently working on creating culturally-tailored education material regarding the steps in the transplant process including explanations of the required tests. The HKTP is working with the hospital system to try and streamline testing into a shorter period of time. The HKTP is also working on improving communication between the transplant center and the referring providers to keep the transplant process moving. Improving living kidney donation is also a priority of the HKTP. The transplant physician from the HKTP has been holding education events in Spanish for the community to discuss kidney donation. Whether these additions improve outcomes will be determined in the future.

Implementing a HKTP appears to be cost effective. Northwestern University implemented a HKTP in 2006 and found that the total costs of the HKTP staffing was < 1.0% per year of the entire transplant center’s annual total costs [23]. In our center, the physician and nursing staff of the HKTP also staff English speaking patients in our CCTCARE. There is one dedicated full time equivalent (FTE) for a Hispanic Spanish-speaking assistant that fully dedicates their time to the HKTP. Thus, the total costs of staffing our HKTP are similar to that of Northwestern University. Creation of a dedicated transplant center with several different ethnic minorities in its catchment area may be more difficult but based on results from our and previous studies, hiring of at least one staff that can dedicate their time to those minority populations may help improve access to transplantation for these populations. This seems like a small cost in order to significantly reduce ethnic disparities in kidney transplantation.

Our study does have limitations. This study was limited to only patients in Colorado that receive their care at UCH. Additionally, while we did find improved patient knowledge in our qualitative analysis, we did not directly measure patient knowledge about kidney transplantation. We did not collect information regarding employment, income or comorbidities and this may have impacted our results. We did not find a significant increase in the percentage of patients listed for transplant. Insurance status, income and comorbidities are all factors that may be reasons patients who were referred and evaluated were disqualified from transplant listing. The COVID-19 pandemic significantly disrupted transplantation in our center in 2020 and then number of transplants performed were significantly decreased which may have affected our results. Notwithstanding these limitations, our study also has several strengths. The use of mixed-methods allowed us to get more detailed information regarding the effectiveness of the HKTP. Trained moderators performed the interviews and the interviewer conducting the patient interviews did not have a clinical relationship with the patients nor was part of the HKTP.

Our study suggests that a tailored clinic to Spanish-speaking patients can improve access to transplantation for Hispanic Americans and leads to increased number of patients referred and evaluated. Future studies need to evaluate whether the HKTP leads to improved number of patients waitlisted and transplanted as well as graft outcomes in Spanish-speaking Hispanic Americans. Additionally, future studies should also focus on improving living donation in Spanish-speaking Hispanic Americans.

## Data Availability

The datasets used and/or analyzed during the current study available from the corresponding author on reasonable request.

## Abbreviations

APPs: Advanced practice providers
CCTCARE: Colorado Center for Transplant Care, Research and Education
DDKT: Deceased donor kidney transplantation
ESKD: End stage kidney disease
HKTP: Hispanic Kidney Transplant Program
LDKT: Live donor kidney transplantation
OPTN: Organ Procurement and Transplantation
UCH: University of Colorado
